# Evidence for functional and regulatory cross-talk between Wnt/β-catenin signalling and Mre11–Rad50–Nbs1 complex in the repair of cisplatin-induced DNA cross-links

**DOI:** 10.18632/oncotarget.27777

**Published:** 2020-11-03

**Authors:** Sanjeev Pasadi, Kalappa Muniyappa

**Affiliations:** ^1^Department of Biochemistry, Indian Institute of Science, Bangalore 560012, India

**Keywords:** cancer, cisplatin resistance, cisplatin-induced DNA crosslinks, Wnt/β-catenin signalling, Mre11–Rad50–Nbs1 complex

## Abstract

The canonical Wnt/β-catenin signalling pathway plays a crucial role in a variety of functions including cell proliferation and differentiation, tumorigenic processes and radioresistance in cancer cells. The Mre11–Rad50–Nbs1 (MRN) complex has a pivotal role in sensing and repairing DNA damage. However, it remains unclear whether a connection exists between Wnt/β-catenin signalling and the MRN complex in the repair of cisplatin-induced DNA interstrand cross-links (ICLs). Here, we report that (1) cisplatin exposure results in a significant increase in the levels of MRN complex subunits in human tumour cells; (2) cisplatin treatment stimulates Wnt/β-catenin signalling through increased β-catenin expression; (3) the functional perturbation of Wnt/β-catenin signalling results in aberrant cell cycle dynamics and the activation of DNA damage response and apoptosis; (4) a treatment with CHIR99021, a potent and selective GSK3β inhibitor, augments cisplatin-induced cell death in cancer cells. On the other hand, inactivation of the Wnt/β-catenin signalling with FH535 promotes cell survival. Consistently, the staining pattern of γH2AX-foci is significantly reduced in the cells exposed simultaneously to cisplatin and FH535; and (5) inhibition of Wnt/β-catenin signalling impedes cisplatin-induced phosphorylation of Chk1, abrogates the G2/M phase arrest and impairs recombination-based DNA repair. Our data further show that Wnt signalling positively regulates the expression of β-catenin, Mre11 and FANCD2 at early time points, but declining thereafter due to negative feedback regulation. These results support a model wherein Wnt/β-catenin signalling and MRN complex crosstalk during DNA ICL repair, thereby playing an important role in the maintenance of genome stability.

## INTRODUCTION

Many chemicals with a mutagenic mode of action interact with DNA, causing changes in its structure; thereby blocking nuclear processes such as DNA replication, transcription, recombination and repair [1]. The widely used anticancer drug cisplatin and its derivatives kill cancer cells by creating intrastrand and/or interstrand cross-links (ICLs) in DNA; thereby inhibit both replication and transcription [2, 3]. Other notable ICL-inducing agents include a variety of endogenous metabolites and exogenous environmental agents that contain bifunctional reactive groups [4]. For example, malondialdehyde (a product of lipid peroxidation) generates adducts in DNA that lead to the formation of ICLs [5].

The insights into the formation of ICLs and their repair have been derived from studies on yeast cells, *Xenopus laevis* oocytes and mammalian cell lines [3, 4]. Specifically, the cells derived from Fanconi anaemia (FA) patient exhibit various types of chromosomal aberrations following an exposure to ICL-inducing agents compared to other genotoxic agents [6]. The cells from bacteria, yeast and mammals remove ICL adducts from their genomic DNA through a complex network of multiple DNA damage response and repair pathways, including mismatch repair, homologous recombination (HR), double strand break (DSB) repair, transcription coupled nucleotide excision repair and base excision repair [7–10]. Regardless of the mechanism involved, the common steps in ICL repair include recognition of ICLs, DNA damage signalling and recruitment of downstream repair proteins. A review of current literature indicates that the Fanconi anaemia pathway of ICL repair involves 22 ‘FANC’ enzymes and accessory proteins; defects in these components cause Fanconi anaemia, a genetic disorder characterized by bone marrow failure and a predisposition to cancer [11, 12]. A growing body of evidences indicates that FA proteins also function in the repair of DNA damage caused by certain types of chemotherapeutic drugs [13, 14].

Among all the DNA lesions, interstrand cross-links are complex lesions that covalently link both strands of an undamaged DNA duplex [2]. Thus, removal of this type of lesion involves unhooking by dual endonucleolytic incisions; consequently, their removal depends on the interplay among different enzymes and accessory proteins of multiple DNA repair pathways [2, 3]. Although intrastrand cross-links can be repaired by nucleotide excision repair, unhooking of interstrand cross-links seemingly occurs during replication-coupled DNA ICL repair [7–10]. The DSBs are common lesions that occur during replication of ICL-containing DNA substrates [15, 16]. The elevated and/or mis-repaired ICLs cause chromosomal breakage and the formation of radial chromosomes, in addition to DSBs [17]. Several studies have demonstrated that the Mre11-Rad50-Nbs1 (MRN) complex, with the support of Sae2 (in yeast) and CtIP (in eukaryotes), helps to preserve genome stability by regulating signalling and repair of DNA damage, HR, controlling the cell cycle checkpoint and maintaining the integrity of telomeres [18, 19]. It has been implied, mostly based on correlative data, that the MRN complex may have a role in DNA ICL repair consistent with its known functions in DNA damage repair [18, 20].

Previous studies have provided evidence that the *MRE11* promoter contains a binding site for β-catenin/LEF heterodimer, the mediator of the canonical Wnt/β-catenin signalling pathway [21]. This pathway governs a myriad of biological processes, including cell fate determination, self-renewal of progenitor cells, adult tissue homeostasis, apoptosis and quiescence [22, 23]. Using genetic and biochemical screens, β-catenin has been identified as a crucial nuclear effector of the Wnt signalling pathway, and several feedback regulatory mechanisms exist to control it [22, 23]. However, the biological effects of Wnt/β-catenin signalling are highly complex as they can be mediated via multiple pathways: aberrant Wnt signalling by either a loss or gain of function is linked with the progression of various diseases, including fibrosis, cancer and Alzheimer’s disease. For example, high Wnt/β-catenin signalling is associated with the upregulation of *LIG4*, which in turn plays a key role in radioresistance in stem cells and cancer cells [24]. Other studies have shown that upregulation of *MRE11* expression through the GSK3β/β-catenin/LEF pathway leads to enhanced DSB repair efficiency in cancer cells [21]. To date, however, little is known about the relationship between Wnt/β-catenin signalling and MRN complex in DNA ICL repair.

Although cisplatin is effective against different types of cancers, the emergence of resistance to cisplatin has become a major challenge in successful cancer treatments [25]. Aberrant DNA repair processes have been implicated in cisplatin-resistance; however, the nature of cellular signalling cues that modulate cisplatin-induced DNA damage is not fully understood. In this study, we use multiple independent assays to investigate the possible relationship between Wnt/β-catenin signalling and components of the cisplatin-induced DNA damage response. Our results reveal that Wnt/β-catenin signalling stimulates the expression of MRN complex subunits, regulates cell cycle progression, increases the number of nuclear γH2AX foci and augments the induction of cell death by cisplatin. Furthermore, Wnt/β-catenin signalling regulates the frequency of HR-based DNA repair. These results support a model wherein Wnt/β-catenin signalling and MRN complex crosstalk during DNA ICL repair, thereby playing a vital role in the maintenance of genome stability.

## RESULTS AND DISCUSSION

### ICL damage stimulates expression of MRN complex subunits in HeLa cells

The Mre11 functions as a dimer and exhibits ATP-dependent 3′–5′ dsDNA exonuclease and ssDNA endonuclease activities, which resect the DNA ends to generate 3′ single-stranded DNA overhangs aided by CtIp1/Sae2 [18, 26, 27]. While Rad50 binds the DNA ends and holds them in close proximity, Nbs1 is essential for nuclear translocation of the Mre11-Rad50 complex; it also interacts with ATM kinase and several other binding partners in DNA damage response [18].

**Figure 1:**
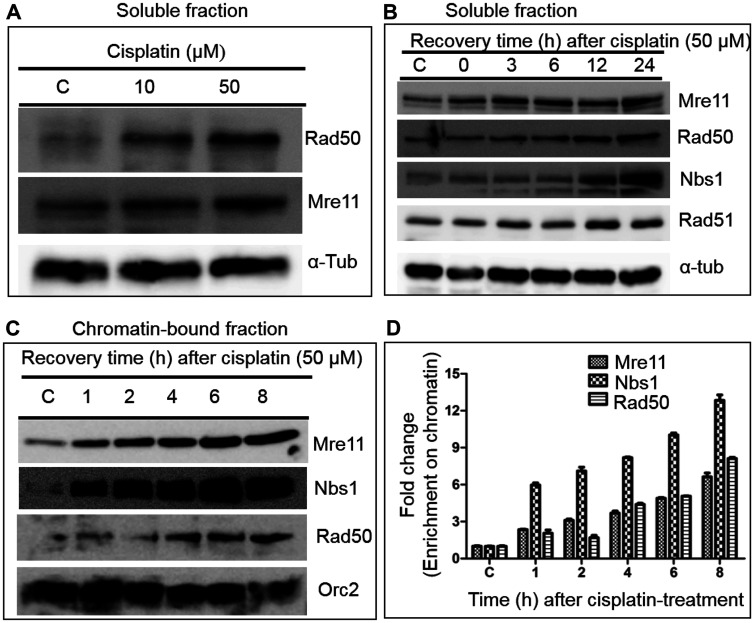
Cisplatin treatment significantly increases the levels of MRN complex subunits in HeLa cells. (**A**) A representative western blot demonstrating the increased levels of Rad50 and Mre11 after cisplatin treatment. (**B**) Kinetics of induction of MRN complex subunits by cisplatin. (**C**) Cisplatin treatment caused a substantial increase in the accumulation of MRN complex subunits in the chromatin-bound fraction. (**D**) Histograms show the mean values of relative level of MRN complex subunits as determined by western blotting and normalized to the corresponding Orc2 band intensities. Alpha-tubulin and Orc2 were used as loading controls for the cytosolic and chromatin fractions respectively. The data is expressed as the mean ± SEM from three independent experiments.

The anticancer activities of cisplatin and its analogues correlate with their ability to cause DNA damage in cancer cells [2]. A key mechanism of cisplatin toxicity is due to its ability to react with the N7 position of guanine to form lethal intrastrand and interstrand cross-links [28]. Although MRN complex is the major sensor of DSBs and is essential for the repair of DNA damage, little is known about its role in human cells treated with cisplatin. A preliminary observation showed that cisplatin induces the expression of MRN complex subunits in the rat kidney [29]; however, its effect on cell viability, expression in human cells and the underlying mechanism remains unknown. As a proof of concept, we first examined cisplatin-induced expression profiles of MRN subunits in HeLa cells by western blot analysis. To normalise the expression levels, and to correct the loading error, α-tubulin was used as an internal control. The levels of Mre11 and Rad50 sharply increased in a dose-dependent manner, indicating that cisplatin induced the expression of these subunits (Figure 1A). The cell viability decreased at doses higher than 50 μM of cisplatin. Accordingly, the cisplatin concentrations utilized in these studies ranged between 10 and 50 μM.

Next, a time course study was conducted of the expression of MRN complex subunits in soluble and chromatin-bound fractions in HeLa cells exposed to 50 μM cisplatin. After cisplatin treatment for 3 h, the cell culture media was changed to fresh media and the cells were allowed to grow. They were harvested at different time points over a 24 h period and the expression profiles of MRN subunits were determined. These time points were chosen because a significant number of cisplatin-treated cells undergo apoptosis at later time points. Interestingly, this analysis revealed that the relative levels of MRN complex subunits sharply increased in both soluble and chromatin-bound fractions in a time-dependent manner (Figure 1B and 1C). To obtain a more robust interpretation, the levels of MRN complex subunits were quantified in the chromatin-bound fraction. The results revealed a 7 to 9-fold enrichment of Mre11 and Rad50, whereas Nbs1 showed an increase of 12-fold at 8 h (Figure 1D). The reason (s) behind the distinct responses of Nbs1 on one hand and Mre11 and Rad50 on the other to cisplatin exposure are unclear but could involve variable thresholds and synergy with other pathways.

Furthermore, a similar expression pattern of MRN subunits was observed in cisplatin-treated U2OS cells (Supplementary Figure 1A). To test the generality of these results, the effect of interstrand cross-linking agent mitomycin C was explored. Remarkably, a significant rise in the levels of Mre11 and Nbs1 was found in HeLa cells treated with increasing concentrations of mitomycin C (Supplementary Figure 1B). These results from two different cell lines are consistent with the idea that DNA ICL agents act through a common mechanism to elicit the expression of MRN complex subunits.

### Effect of Wnt/β-catenin signalling modulators on cytotoxic effects of cisplatin

The increased abundance of MRN complex subunits in cisplatin and mitomycin C-treated HeLa and U2OS cells favour the notion that the MRN complex may play a key role in ICL-induced DNA double-strand breaks. Furthermore, accumulating evidence suggests a crosstalk between the cellular DNA repair machinery and DNA damage signalling pathways [30, 31]. In this regard, several studies have shown that the perturbation of Wnt/β-catenin signalling alters physiological processes leading to cancer [32, 33], and activation of the Wnt/β-catenin signalling induces radioresistance in several human cancer cells [21]. A wide range of small molecule Wnt/β-catenin signalling modulators have been developed, because of the possibility that targeting the Wnt/β-catenin signalling pathway has implications in anticancer therapy [34, 35]. To that end, two small molecules, namely CHIR99021 (henceforth referred to as CHIR for simplicity) and FH535, were employed to investigate the potential relationship between Wnt/β-catenin signalling and ICL repair. The former potentiates the Wnt/β-catenin signalling through the inhibition of GSK3β (which blocks the degradation of β-catenin), while the latter inhibits it by attenuating the β-catenin/TCF complex formation. These have been shown to effectively modulate Wnt/β-catenin signalling in cancer models [36, 37]. Additionally mirin, a potent inhibitor of Mre11 exonuclease activity [38] was used to validate and optimize the effects of Wnt/β-catenin signalling modulators.

**Figure 2:**
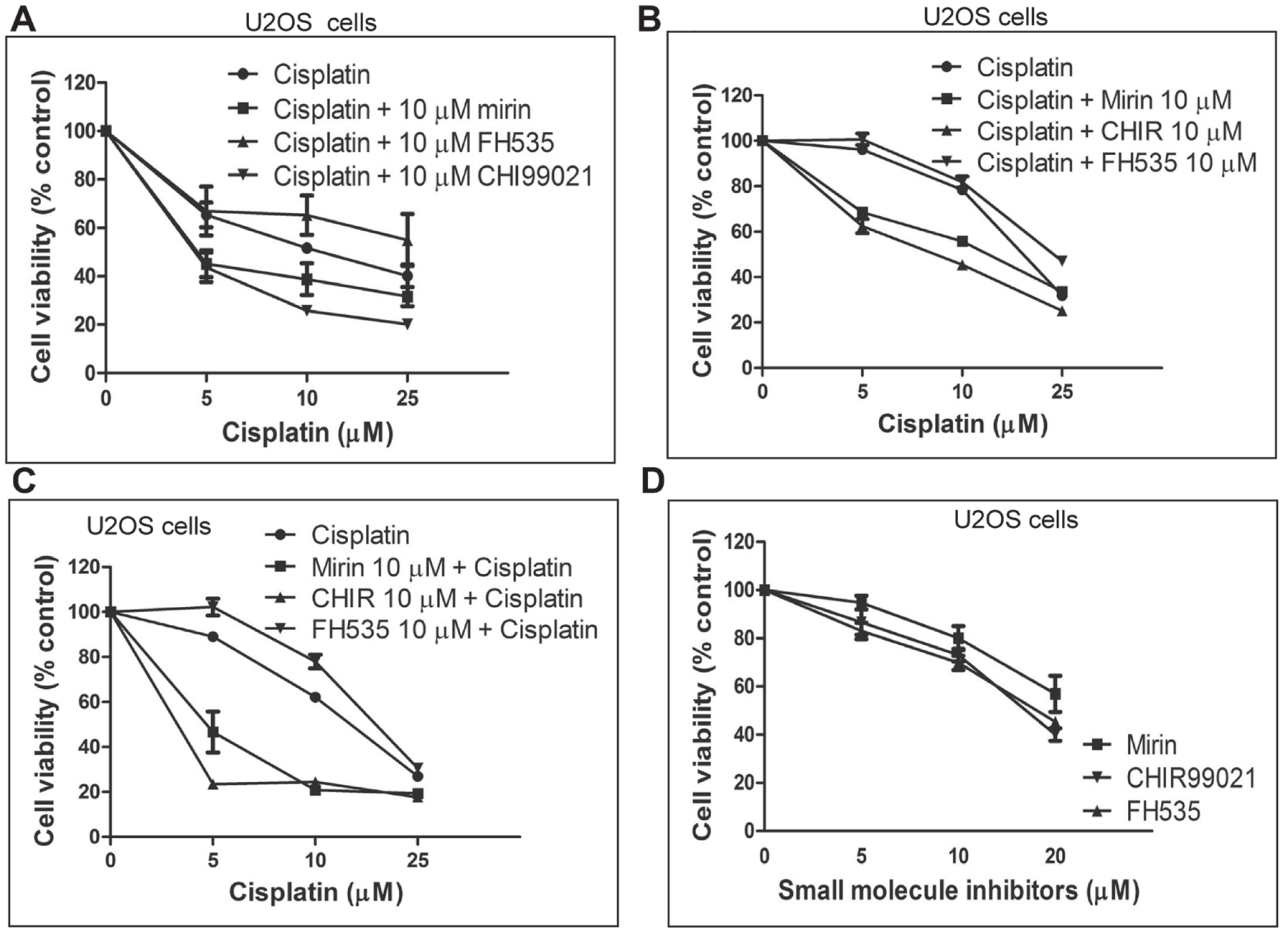
Wnt/β catenin signalling has adverse effects on the viability of U2OS cells following cisplatin treatment. (**A**) The effect of cisplatin alone or in combination with mirin or Wnt modulators on the viability of U2OS cells. The cells were treated with the indicated doses of cisplatin for 3 h and recovered for a period of 3 days in a fresh medium containing 10 μM of mirin, FH535 or CHIR. (**B**) Same as (A), except that the cells were monitored for a period of 10 days. (**C**) The cells were pre-treated for 24 h with the indicated Wnt modulator before adding cisplatin. The MTT assay was performed after a period of 10 days. (**D**) Effect of mirin, FH535 or CHIR on the viability of U2OS cells. The standard deviations were derived from three independent experiments.

Thus, we sought to explore whether the Wnt/β-catenin signalling modulators can affect cisplatin-mediated cytotoxicity using the MTT cell viability. For this purpose, cells were first exposed to different doses of cisplatin, and then to a sub-lethal concentration (10 μM) of CHIR or FH535. Our results show that exposure to cisplatin induced cytotoxicity in U2OS cells, in a concentration-dependent manner with an LC_50_ value of 17.1 μM (Figure 2A, Supplementary Table 1). A resistance to the cytotoxic effects of cisplatin was manifested by a plateau at the higher doses, possibly due to the arrest of downstream apoptotic pathways. To determine the effects of CHIR and FH535 on cisplatin-induced cytotoxicity, the U2OS cells were treated with increasing concentrations of cisplatin. After removal of cisplatin-containing media, fresh media was added to cells along with 10 μM CHIR, FH535 or mirin and incubated for the indicated time periods. Interestingly, the results show that CHIR potentiated cisplatin-induced cell death and resulted in a reduction of the LC_50_ value of cisplatin to 8.96 μM. Similarly, mirin displayed synergistic cytotoxic effects with cisplatin in U2OS cells. In contrast, cisplatin-induced cell toxicity was partially rescued when the cells were treated with FH535 (Figure 2A, Supplementary Table 1).

To confirm these results, cell viability assays were carried out using two different approaches. In the first approach, the cell viability was monitored over a 10-day period. In the second approach, the cells were pre-treated with mirin or Wnt/β-catenin modulators for 24 h before cisplatin addition. After 3 h of incubation with cisplatin, the cells were re-seeded in fresh media and grown in the presence of Wnt/β-catenin modulators over a 10-day period. Both approaches revealed that co-incubation with CHIR and cisplatin reduced the cell viability and lowered the LC_50_ values of cisplatin (Figure 2B and 2C). On the other hand, the attenuation of Wnt/β-catenin signalling by FH535 augmented cell viability and also increased the LC_50_ value of cisplatin. The exposure of cells to cisplatin and mirin led to a further decrease in cell viability and also reduced the LC_50_ value of cisplatin to 16.05 μM (Supplementary Table 2). In the second approach, the cells were pre-treated with Wnt/β-catenin modulators or mirin for 24 h, before exposure to cisplatin. It was noticed that both mirin and CHIR hypersensitized the cells and, consequently reduced the LC_50_ values to 8.5 and 6.4 μM respectively (Supplementary Table 3). In parallel, it was seen that the Wnt/β-catenin modulators or mirin by themselves caused cytotoxicity, albeit at relatively high concentrations (Figure 2D). Altogether, these results are consistent with the idea that Wnt/β-catenin signalling plays a key role in the regulation of ICL repair.

### Impairment of cell cycle progression by Wnt/β-catenin signalling modulator CHIR

**Figure 3:**
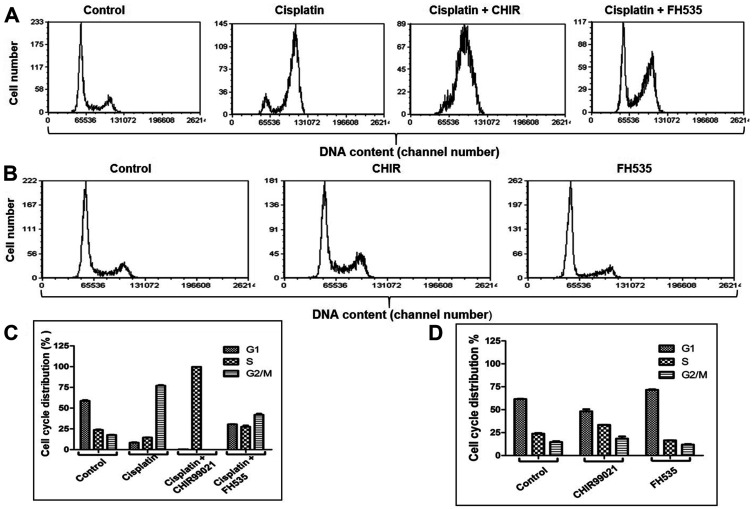
Inhibition of the Wnt/β-catenin pathway attenuates cisplatin induced cell cycle arrest. (**A**) Treatment with cisplatin, cisplatin and CHIR or cisplatin and FH535 alter cell cycle regulation in U2OS cells. (**B**) Cell cycle profiles of U2OS cells treated with CHIR or FH535. (**C** and **D**) Show quantification of the percentage of G1, S and G2/M phase cells, determined from (A and B). The data are expressed as the mean ± SEM from three independent experiments.

The cell cycle checkpoints and surveillance mechanisms arrest cells at various stages of the cell cycle to promote DNA repair or cell death in the case of non-repairable damage [39]. The cancer cells are often compromised with the surveillance and checkpoint mechanisms [40]. To investigate the potential role of Wnt/β-catenin signalling modulators in ICL repair, a cell cycle analysis was carried out using FACS following ICL induction. The U2OS cells exposed to cisplatin alone and cisplatin together with CHIR or FH535 were cultured for 36 h. The cells were stained with propidium iodide prior to FACS analysis and the proportion of cells in the G1, S and G2/M phases of the cell cycle was estimated using the FCS Express software. The results showed that cisplatin causes cell cycle arrest at the G2/M phase. The percentage of cells in each cell cycle phase (G1, S and G2/M) was 8.41%, 14.54%, and 77.05%, respectively (Figure 3A and 3C). Interestingly, an exposure of the cells to cisplatin in combination with CHIR resulted in an accumulation of > 99% of the cells in the S phase (Figure 3A and 3C). However, co-treatment of cells with cisplatin and FH535 significantly attenuated the action of cisplatin, resulting in the depletion of G2/M population of cells (Figure 3A). The proportion of cells in the G1, S and G2/M phases was 30.48%, 27.59%, and 41.92%, respectively (Figure 3C). These results imply that the effects exerted by FH535 were roughly equal through all phases of the cell cycle. On the other hand, the distribution of cells treated individually with FH535 or CHIR showed less striking differences compared to the untreated control cells, although their effects become more pronounced when combined with cisplatin (Figure 3B and 3D; Supplementary Tables 4 and 5). Altogether, these data suggest a functional link between Wnt/β-catenin signalling and cisplatin-induced DNA damage repair.

### CHIR potentiates the formation of cisplatin-induced γH2AX foci

**Figure 4:**
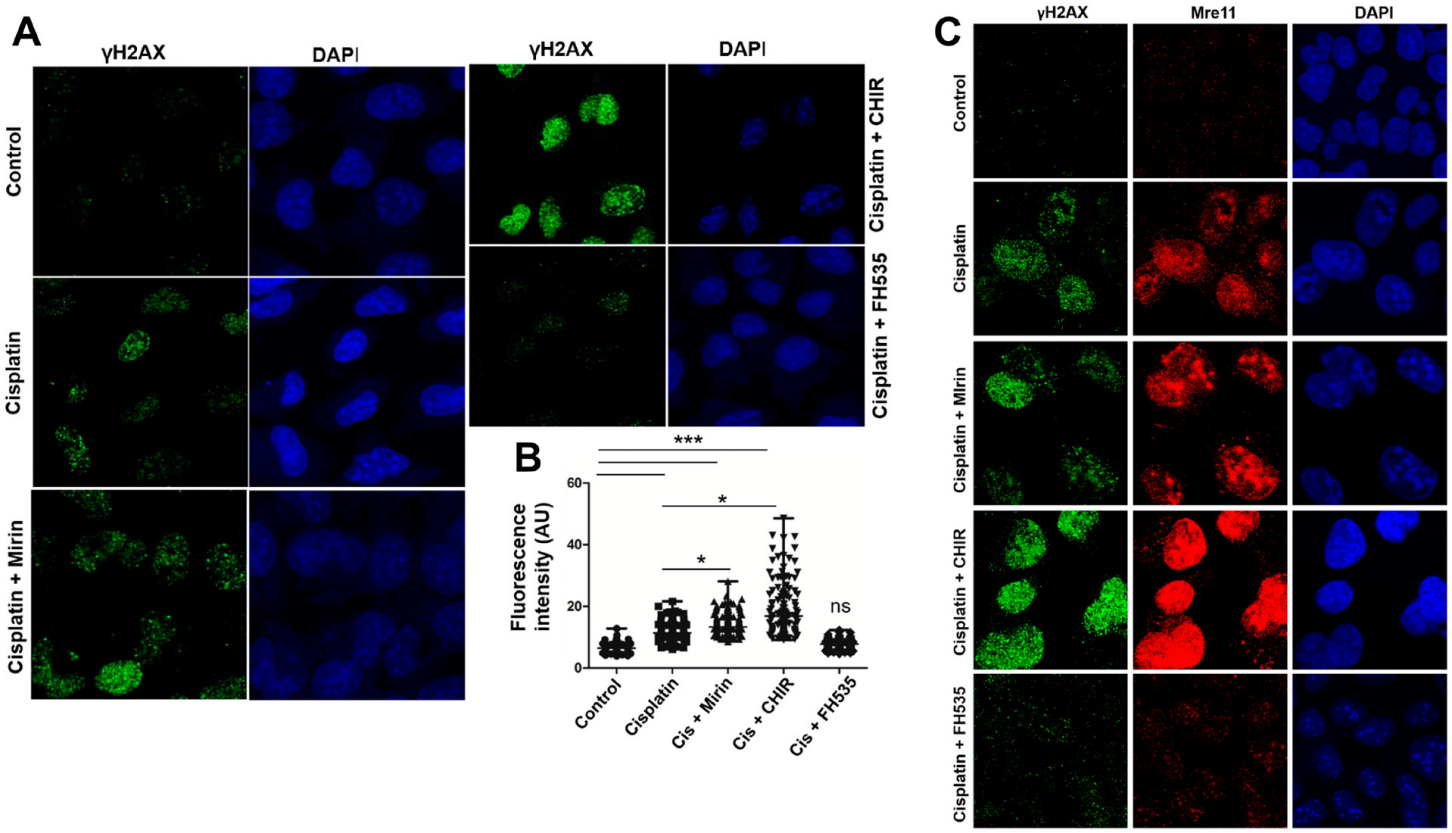
Modulation of Wnt/β-catenin pathway impairs the efficiency of DNA repair. (**A**) Visualization of γ-H2AX nuclear foci in the cell line U2OS. The cells were treated with cisplatin alone, mirin and cisplatin or cisplatin plus CHIR or FH535. (**B**) Scatter plot of γH2AX foci-positive cells following treatment with different chemotherapy agents. A minimum of 100 cells were counted for each sample. The graph is a representative of three independent experiments. (**C**) Visualization of foci of γ-H2AX in relation to Mre11 in the cell line U2OS. The cells were treated with cisplatin alone, cisplatin + Mirin or cisplatin plus CHIR or FH535. The qualitative assessment was done on confocal microscope (Magnification ×60); scale bar, 20 μm.

Previous studies have revealed that γH2AX plays an important role in cellular response to DNA damage and is required for the assembly of DNA repair proteins [41]. Therefore, γH2AX foci have been widely used as surrogate markers for DNA damage throughout the genome consequent to DSB formation [42]. To test this possibility, γH2AX-foci were scored to assess the effect of Wnt/β-catenin signalling modulators on cisplatin-induced pan-nuclear γH2AX-foci in U2OS cells. Consistent with data from the MTT assay and cell cycle analysis, a visual inspection of the images indicated significant differences in the size and distribution of γH2AX foci between untreated control and treated cells (Figure 4A). In treated cells, the γH2AX foci appeared to be randomly distributed across the cell nucleus, while the untreated control cells had no detectable γH2AX-positive foci (Figure 4A, left side, upper panel). Furthermore, cells that were exposed to cisplatin had significantly greater numbers of γH2AX foci per cell (Figure 4A, left side, middle panel). This result is consistent with the DNA-damaging effects of cisplatin [43]. By comparison, the number of γH2AX foci further increased in the cells treated with a combination of cisplatin and mirin (Figure 4A, left side, lower panel). Interestingly, the number of cells with intensely-stained γH2AX foci markedly increased when the cells were exposed simultaneously to cisplatin plus CHIR (Figure 4A, right side, upper panel). By contrast, FH535 (inhibitor of Wnt/β-catenin signalling) completely abolished the appearance of γH2AX foci due to attenuation of cisplatin-induced DSBs (Figure 3A, right side, lower panel). These results are also supported by quantification of γH2AX foci per cell. The median fluorescence intensity of γH2AX foci was 2-fold and 2.5-fold higher in cells exposed to cisplatin and mirin or cisplatin and CHIR, respectively (Figure 4B). A 1.7-fold increase in γH2AX foci was found in cisplatin-treated cells compared to untreated cells.

Next, we sought to determine the possible correlation between the appearance of Mre11 foci in relation to γH2AX foci in U2OS cells treated with cisplatin alone or cisplatin in combination with mirin or Wnt/β-catenin signalling modulators. The cisplatin-treated cells showed discrete Mre11 foci (Figure 4C, row 2), which markedly increased in cells exposed simultaneously to cisplatin plus mirin or cisplatin and CHIR (Figure 4C, row 3 and 4). In contrast, the Mre11 foci were almost absent in cells exposed simultaneously to cisplatin plus FH535 (Figure 4C, row 5). These results suggest a direct correlation between DSB induction and an increase in the numbers of γ-H2AX and Mre11 foci per cell.

### Wnt/β-catenin signalling modulators suppress gene conversion between sister chromatids

To further dissect the functional consequences of deregulation of the Wnt/β-catenin signalling pathway, a previously described reporter assay was used to determine the long tract gene conversion events arising between sister chromatids [44]. The gene conversion between tandem mutant copies of the chromosomally integrated *gfp* gene in transfected U2OS cells was induced by transient expression of the site-specific endonuclease I-SceI. The possible role of Mre11 in the gene conversion events was explored in the context of Wnt/β-catenin signalling modulators alone or in combination with mirin. Mirin inhibits the cellular pathways mediated by Mre11 nuclease *in vivo* including the DNA end-resection step of HR [46]. On the other hand, LiCl decreases the activity of GSK-3β [45].

**Figure 5:**
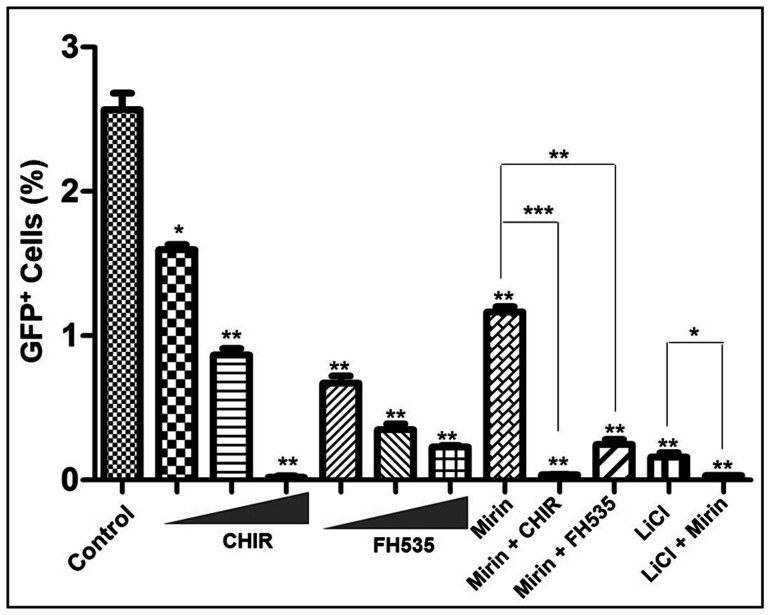
The modulators of Wnt/β-catenin signal transduction pathway alter the efficiency of HR. The triangle on the x-axis represents increasing concentrations (10, 25 and 50 μM) of CHIR or FH535. Mirin was used at 25 μM concentration. For combinatorial treatments, the cells were treated with 25 μM each of mirin, CHIR and FH535, while concentration of LiCl was at 25 mM. The small molecule modulators and inhibitors were added to the cells 6 h post transfection. The data are expressed as the mean ± SEM from three independent experiments. The statistical significance was determined using Student’s *t* test. ^*^ *p* < 0.05, ^**^ *p* < 0.01, ^***^ *p* < 0.001.

The results indicate that the DSB repair by gene conversion is robust in untreated U2OS cells (Figure 5). To clarify the roles of Mre11 and Wnt/β-catenin signalling, we measured the frequencies of gene conversion in the presence of indicated effector molecules under similar conditions. This analysis showed a linear correlation between the percentage of GFP-positive cells and the concentration of CHIR. As the amount of CHIR increased, the frequency of gene conversion sharply decreased by 128-fold, indicating a dose-dependent quantitative response. To analyse this correlation further, we tested the contributing effect of FH535, a small-molecule inhibitor of the Wnt/β-catenin signalling pathway. Intriguingly, FH535 suppressed the gene conversion, albeit at a very low dose. This was unexpected because FH535 renders protection against cisplatin-induced cytotoxicity; further studies will be needed to clarify the underlying mechanisms. Consistent with previous studies [46], an exposure of the cells to mirin led to 2.2-fold decrease in GFP-positive cells (Figure 5). However, mirin, in combination with CHIR, LiCl or FH535, showed a greater decrease in the yields of GFP-positive cells than mirin alone, indicating that both Mre11 and the Wnt/β-catenin signalling pathway act synergistically in the gene conversion events between sister chromatids.

### Wnt signalling modulators regulate the expression of AXIN2 and MRE11

A number of switchable transcription factors regulate the transcription of Wnt/β-catenin protein-coding target genes [47]. To further explore the relationship between Wnt/β-catenin signalling and cisplatin-induced DSBs, we investigated the expression of two target genes of the Wnt signalling pathway. The established Wnt target gene *AXIN2* is a negative feedback regulator of the canonical Wnt/β-catenin signalling pathway, and its gene product promotes the phosphorylation and degradation of β-catenin [47, 48]. The expression levels of *AXIN2* mRNA and protein are up-regulated by the activation of the Wnt/β-catenin pathway, indicating that *AXIN2* is a direct target of the Wnt pathway, mediated through TCF/LEF factors [48].

**Figure 6:**
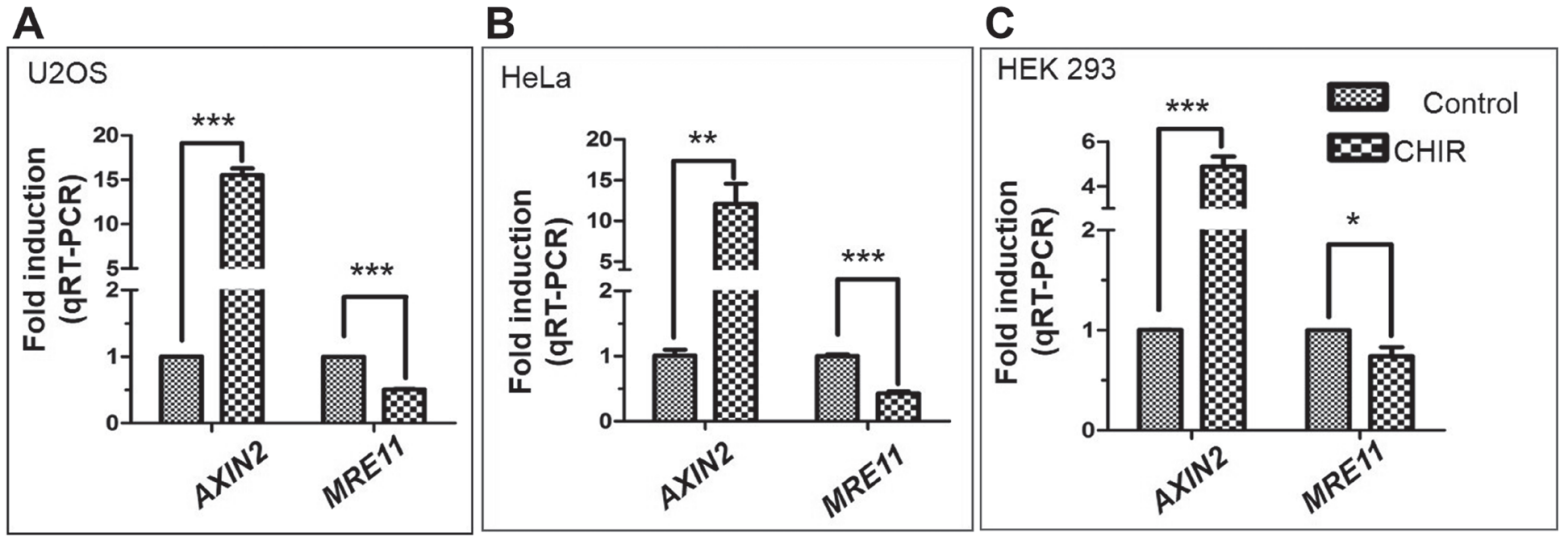
Differential expression profiles of MRE11 and AXIN2 genes following treatment of human cancer cell lines with CHIR (25 μM). RT-qPCR showing mRNA levels of *AXIN2* and *MRE11* in (**A**) U2OS; (**B**) HeLa and (**C**) HEK293 cells. The normalization factor calculation is based on the geometric mean of relative amounts of three control genes: *GAPDH*, *HPRT* and *TBP*. The standard deviations were derived from three independent experiments. The statistical significance was determined using Student’s *t* test. ^*^ *p* < 0.05, ^**^ *p* < 0.01, ^***^ *p* < 0.001.

We hypothesised that the downstream target molecules of the Wnt/β-catenin signalling pathway might be overexpressed in the cells exposed to CHIR. To test this premise, the expression of *AXIN2* in U2OS cells was monitored by qRT–PCR. Our results revealed a strong upregulation of *AXIN2* gene expression in the cells exposed to CHIR as compared to untreated cells. Surprisingly, a decrease in the *MRE11* mRNA levels was observed (Figure 6A). This is in contrast with the results of a previous study [21], which showed that β-catenin/LEF-1 upregulates *MRE11* expression. Prompted by the above results and to assess the generality of the findings, the expression profile of *AXIN2* was investigated in different cell lines. Consistent with the observation in U2OS cells, the *AXIN2* expression was upregulated in both HeLa and HEK293 cell lines exposed to CHIR. In contrast, the *MRE11* expression declined in these cells (Figure 6B and 6C). It is interesting to note that the levels of *AXIN2* mRNA expression were significantly higher in CHIR-treated U2OS and HeLa cells than that in HEK293 cells.

### Cisplatin treatment activates Wnt/β-catenin signalling

**Figure 7:**
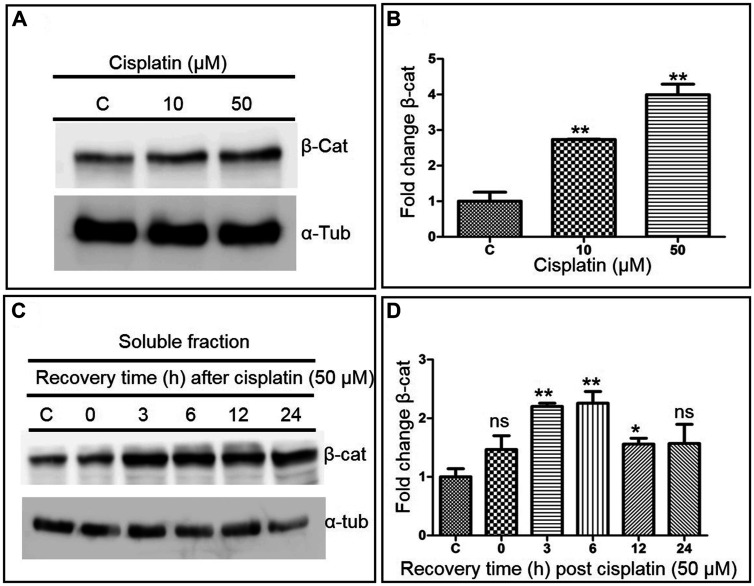
ICL damage activates Wnt/β-catenin signalling. (**A**) Representative western blot reflecting β-catenin levels in U2OS cells following treatment with increasing doses of cisplatin. (**B**) Histograms showing the relative changes in β-catenin levels in cisplatin treated cells, determined from (A). (**C**) Representative western blot showing the kinetics of induction of β-catenin by cisplatin in U2OS cells. (**D**) The histograms shows the relative changes in β-catenin levels in the cytosolic fraction of cisplatin treated cells, determined from (C). The data is expressed as the mean ± SEM from three independent experiments. The statistical significance was examined using Student’s *t* test. ^*^ *p* < 0.05, ^**^ *p* < 0.01, ns, not significant.

In unstimulated, resting cells, β-catenin is phosphorylated at Ser33, Ser37 and Thr41 by GSK3; then, it is polyubiquitinated by β-TrCP and targeted to proteasomal degradation [49, 50]. β-catenin is protected from degradation upon activation of the canonical Wnt signalling pathway [47, 48]. Upon entering the nucleus, the newly-stabilized β-catenin acts as a transcriptional co-activator for TCF/LEF to activate the Wnt target genes [47, 48]. To explore the link between suppression of gene conversion and Wnt signalling in cells treated with cisplatin, the expression of β-catenin was analysed. It was observed that the β-catenin levels increased by 3 to 4-fold in U2OS cells treated with cisplatin in a dose-dependent manner as compared to untreated cells (Figure 7A and 7B). The kinetics of β-catenin expression after cisplatin treatment showed that its levels increased steadily up to 6 h and declined thereafter (Figure 7C and 7D). To further confirm these results, we tested the effect of another ICL inducing agent, mitomycin C, on Wnt signalling. The western blotting analysis showed that the β-catenin levels increased by 3 to 5-fold in cells treated with mitomycin C compared to untreated cells (Supplementary Figure 2). Altogether, these findings support the notion that ICL inducing agents activate the Wnt signalling pathway.

### Inhibition of Wnt/β-catenin signalling pathway leads to defects in checkpoint control in cisplatin-treated U2OS cells

In response to DNA damage, phosphorylation of Chk1 on S345 activates the G2/M checkpoint to delay progression of the cell cycle in order to ensure DNA repair [51–53]. The phospho-Chk1 is also essential for cell cycle checkpoint during the intra S phase [54–56]. Cisplatin induces G2-phase cell cycle arrest, premature cell senescence and apoptosis [57, 58]. The findings described above suggest that ICL agents activate the canonical Wnt signalling pathway as evidenced by the elevated β-catenin levels, indicating that Wnt signalling plays a crucial role in ICL repair. In addition, inhibition of Wnt signalling affected the HR efficiency. These observations posit that an increase in cell viability is likely due to the defects in mitotic cell cycle checkpoint arrest. Accordingly, phospho-Chk1 was used as a marker to monitor the cisplatin-induced cell cycle arrest. In addition, the relationship between canonical Wnt/β-catenin signalling and DNA damage-induced activation of Chk1 was examined using Wnt modulators, namely CHIR99021 and FH535, to activate and repress Wnt/β-catenin signalling respectively in cisplatin-treated U2OS cells.

**Figure 8:**
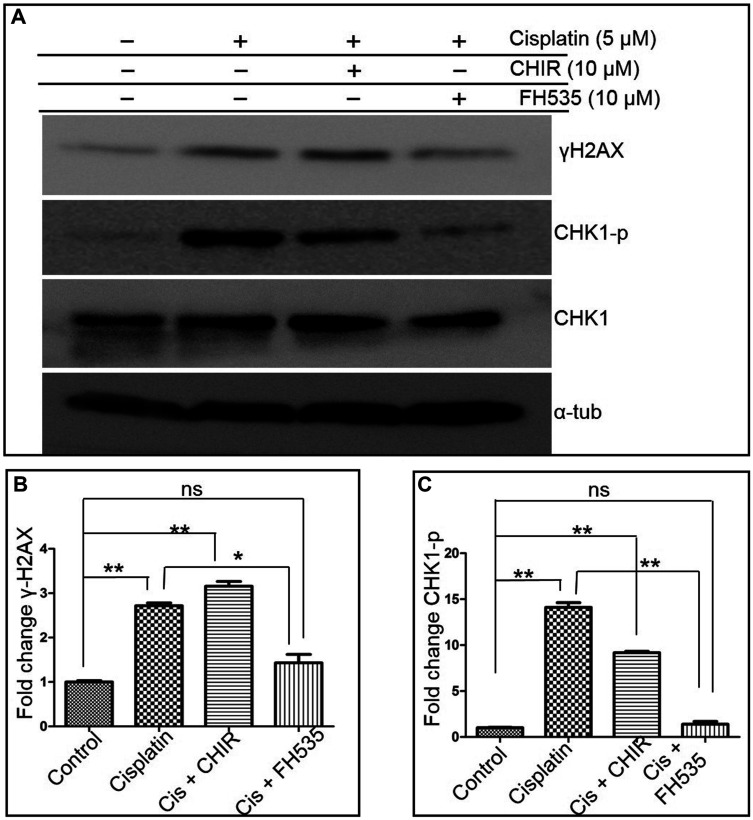
FH535 abrogates cell cycle checkpoint activation. (**A**) Representative western blots showing the levels of γ-H2AX, Chk1p (phosphorylated at S345), Chk1 and α-tubulin. (**B** and **C**): histograms showing the relative levels of γ-H2AX and Chk1p. The intensities of the bands in (A) were quantified and normalized to the corresponding α-tubulin band intensities. The data is expressed as the mean ± SEM from three independent experiments. The statistical significance was determined using Student’s *t* test. ^*^ *p* < 0.05, ^**^ *p* < 0.01; ns, not significant.

We found basal levels of phospho-Chk1 (due to constitutive phosphorylation) and γH2AX in untreated cells (Figure 8A and 8B). In contrast to the 3-fold increase in the level of γH2AX (indicating DNA damage), a dramatic 15-fold increase in phospho-Chk1 was seen following cisplatin treatment, consistent with a role for Chk1 in DNA damage response (Figure 8A and 8C). Interestingly, the phospho-Chk1 levels reduced in cells treated with a combination of cisplatin and CHIR or cisplatin and FH535 (Figure 8C). Moreover, the relative efficacy of these modulators was different: while CHIR partially decreased the levels of phospho-Chk1, FH535 almost completely blocked the cisplatin-induced phosphorylation of Chk1. These results suggest that the reduced HR observed in cells treated with FH535 resulted from the decreased cell cycle arrest at the G2/M phase and DNA repair by HR. These findings are in accord with previous studies that report that Chk1 knockdown abrogates *RAD51* foci formation, consistent with the notion that Chk1 is important for DNA repair [59].

### Differential regulation of Wnt/ β-catenin signalling by small molecule modulators

A hallmark of the canonical Wnt/β-catenin signalling pathway is the activation of β-catenin. Consequently, the β-catenin/LEF-1 heterodimer binds to the promoter regions of specific target genes, including *MRE11*, and upregulates their expression [21]. Moreover, Mre11 and FANCD2 have been shown to be required for ICL repair; also, the MRN complex is a critical regulator of FANCD2 stability and function during DSB repair [60]. To better define the regulation of canonical Wnt signalling by small molecule modulators, we sought to elucidate the gene expression profiles of β-catenin, Mre11 and FANCD2. In a series of experiments, CHIR-treated U2OS cells were harvested at the indicated time points and the whole-cell lysates were subjected to SDS-PAGE followed by western blot analysis with the indicated antibodies.

**Figure 9:**
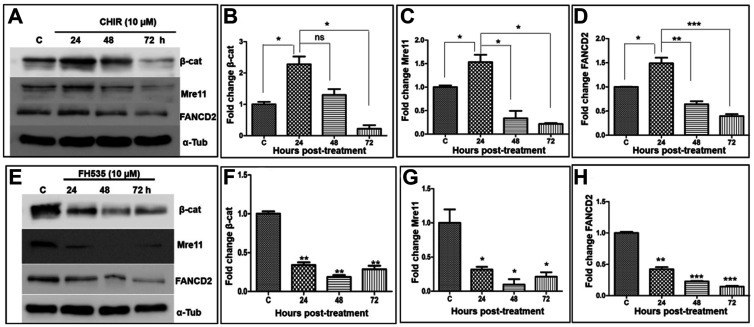
Wnt modulators affect the expression of β-catenin, Mre11 and FANCD2. (**A**) Kinetic analysis of CHIR-mediated regulation of the expression of β-catenin, Mre11 and FANCD2. (**B**–**D**): histograms showing the quantification of the relative levels of β-catenin, Mre11 and FANCD2 respectively, determined from (A). (**E**) Kinetics of suppression of β-catenin, Mre11 and FANCD2 expression by FH535. (**F**–**H**): histograms showing the quantification of the relative levels of β-catenin, Mre11 and FANCD2 respectively, determined from (E). Alpha-tubulin was used as an internal control. The data is expressed as the mean ± SEM from three independent experiments and normalized to α-tubulin levels. The statistical significance was examined using Student’s *t* test. ^*^ *p* < 0.05, ^**^ *p* < 0.01, ^***^ *p* < 0.001, ns, not significant.

Our analysis revealed that the β-catenin levels rose by ~2.5-fold at 24 h in CHIR-treated cells compared to the untreated cells and gradually declined below its basal level at 72 h (Figure 9A and 9B). Mirroring the levels of β-catenin, a similar expression pattern was observed with Mre11 and FANCD2 in response to CHIR treatment (Figure 9A, 9C, and 9D). It should be noted that the decreased β-catenin expression cannot be attributed to the CHIR-induced cytotoxic effects. Several studies have established that Wnt/β-catenin signalling is regulated by the Wnt negative-feedback regulation of target genes such as *AXIN2* and *DKK1* [48, 61–64]. Therefore, these findings raise the possibility that the decreased levels of β-catenin is likely due to a negative feedback loop mediated by *AXIN2* through the degradation of β-catenin. This premise was validated by testing the effect of CHIR on *AXIN2* expression, which is directly responsible for formation of the β-catenin destruction complex. Remarkably, a time-dependent and robust up-regulation of *AXIN2* mRNA was seen following CHIR treatment (Supplementary Figure 3), indicating that the reduced levels of β-catenin at later time points is due to a negative feed-back mechanism.

We next investigated the effect of FH535, which is a synthetic small molecule that antagonizes the action of β-catenin/TCF/LEF transcription factors [37, 65]. Our data suggest that FH535 attenuates β-catenin expression by 2-fold compared to untreated cells (Figure 9E and 9F). Similarly, a significant decrease in Mre11 and FANCD2 levels was seen, suggesting that Wnt signalling regulates the expression of Mre11 and FANCD2 (Figure 9G and 9H). Together, these results indicate that the expression of downstream target genes of Wnt signalling is inversely regulated by CHIR and FH535. To further confirm the regulation by Wnt modulators, we quantified the expression profiles of β-catenin, Mre11 and FANCD2 following exposure to different concentrations of CHIR or FH535. It was found that the levels of β-catenin, Mre11 and FANCD2 correlated well with the CHIR concentrations after 24 h of culture (Supplementary Figure 4A–4D). Conversely, an inverse correlation was found between the levels of β-catenin, Mre11 and FANCD2 expression and the concentrations of FH535 (Supplementary Figure 4E–4H). Collectively, these results reveal a concentration-dependent effect of CHIR or FH535 on downstream target genes.

Previous studies have established that the deregulation of canonical Wnt/β-catenin pathway is associated with colorectal cancer, as well as other cancers, such as gliomas [66, 67]. Accordingly, we addressed whether the expression of MRE11 is affected in the glioblastoma cell lines LN229 and U87. Western blot analyses were carried out to assess alterations in the protein levels of Mre11 and β-catenin, using α-tubulin as an internal control. A significant decrease in Mre11 protein levels was seen in the cell line LN229 compared to the cell line U87 (Supplementary Figure 5A and 5B). On the other hand, the protein levels of β-catenin were not altered in these cell lines. The gene expression profiles of *MRE11, CTNNB1* (β-catenin) and *CCND1* (cyclin D1; a bona fide Wnt target gene) were assessed by real-time qPCR. The data reveal that MRE11 and CCND1 mRNA levels were significantly downregulated in the cell line LN229 compared to the cell line U87 (Supplementary Figure 5C), indicating a crosstalk between the Wnt/β-catenin signalling pathway and MRN complex.

## MATERIALS AND METHODS

### Cell lines and culture conditions

HeLa, U2OS and LN229 cells lines were cultured in Dulbecco’s Modified Eagle Medium (DMEM; Sigma-Aldrich, St. Louis, USA) supplemented with 10% FBS (Invitrogen Life Technologies, Carlsbad, CA) at 37°C in humidified air containing 5% CO_2_. The cell lines were verified through microscopic examination of their morphological properties and testing for mycoplasma by Hoechst staining.

### Cell viability assay

Typically, ~2000 cells were grown in a 96-well plate in Dulbecco’s Modified Eagle Medium (DMEM) (Sigma-Aldrich, St. Louis, MO, USA) supplemented with 10% FBS at 37°C in humidified air containing 5% CO_2_. The cells after reaching 70% confluency were treated with the indicated concentrations of cisplatin (Sigma-Aldrich). After a wash, the cells were allowed to recover in the fresh medium. The cells were further cultured in DMEM containing 10% FBS with different concentrations of CHIR990021 (henceforth CHIR), mirin or FH535 (Sigma-Aldrich). In other assays, the cells were pre-treated with Wnt modulators for 24 h before cisplatin treatment. After 3 h with cisplatin, the cells were recovered in a fresh medium and incubated with CHIR or FH535. The cell viability after short-term (3 days) and long-term (10 days) exposures to cisplatin were determined by an MTT assay as described [68]. The relative cell viability (%) was computed as follows: (A_570_ of treated samples/A_570_ of untreated samples) × 100.

### Cell cycle analysis

The Wnt modulators (CHIR, FH535) or dimethyl sulfoxide were added to the cultures, which had been exposed to cisplatin for 3 h and recovered in a fresh medium. After incubation for 36 h, the cells were harvested by well-trypsinization and fixed with 70% ethanol for 12 h at –20°C. After filtration through a 40 micron-cell strainer, the cells were centrifuged and incubated with 0.1 mg/ml RNase A in phosphate buffered saline (PBS) for 3 h at 42°C. The cells were stained with propidium iodide solution (50 μg/ml) in the dark on ice for 5 min. The DNA content of each cell preparation (~10000 cells) was analysed by a FACSVerse flow cytometer (BD Biosciences, San Jose, CA, USA) as described [69]. The aggregated cells were gated out and the percentage of cells with 2N and 4N DNA content was determined using the FCS Express 4 software.

### Microscopy, image acquisition, and analysis

The cells from the exponential growth phase were seeded onto round glass coverslips (diameter of 18 mm). After 24 h, the cells were exposed to cisplatin (10 μM) for 5 h at 37°C; then, they were treated for 24 h with 25 μM of CHIR, FH535 or mirin at 37°C. The cells were fixed in PBS containing 4% formaldehyde and permeabilized with 90% methanol for 10 min at 24°C. The cells were washed with PBS and blocked for 30 min in PBS containing 0.5% bovine serum albumin and 0.5% Triton X-100. After incubation with γH2AX antibody (1:2000) for 2 h, the cells were incubated with fluorescein isothiocyanate (FITC)-conjugated secondary antibody (1:100). In the case of Mre11, cells were incubated with Mre11 antibody (1:1000) for 2 h and incubated with TRITC-conjugated secondary antibody (1:100). After 1 h at 24°C, the cells were stained with DAPI (4ʹ, 6-diamidino-2-phenylindole) for 5 min at 24°C. The cells were washed four times with PBS and examined using a Leica TCS SP5 Confocal Imaging System (Leica Microsystems, Wetzlar, Germany). The images were processed using the LAS-AF-Lite 2.4.1 software (Leica Biosystems) and quantified using the imageJ software. A minimum of 100 cells were used for quantification of γH2AX foci per sample.

### RNA isolation and quantitative real-time PCR

Total RNA was isolated from lysed cells using TRI reagent (Sigma-Aldrich). One mL of cell homogenate was mixed with 0.2 ml chloroform. After incubation for 10 min at 24°C, the suspension was centrifuged at 13000 rpm for 10 min at 4°C. The aqueous phase was transferred to a clean tube and the RNA was precipitated with 2-propanol. The pellet was washed with 70% ethanol and resuspended in 20 μl of RNAase free water. The samples were then treated with DNase I to remove any genomic DNA contamination. The RNA (1 μg) was reverse transcribed using the iScript cDNA synthesis kit (Bio-Rad Laboratories, CA, USA). The cDNA was quantified using the iTaq universal SYBR green supermix kit (Bio-Rad Laboratories, CA, USA). The forward and reverse primers were used to amplify *MRE11* cDNA (5ʹ-TAGCATCTCAGCAGCAACCA-3ʹ and 5ʹ-TTTAAAGGCTCTTCCTCTTTGAGAC -3ʹ) or *AXIN2* cDNA (5ʹ-CTTGACCCTGGGCCACTTTA-3ʹ and 5ʹ-ATCTCCTCAAACACCGCTCC-3ʹ), respectively. The following reference genes were used to normalize the qPCR data: *GAPDH, HPRT*, and *TBP*. Their cDNA was amplified using the forward and reverse primers, respectively; *GAPDH* (5ʹ-GTCATCCCTGAGCTGAACGG-3ʹ and 5ʹ-GG CAGGTTTTTCTAGACGGC-3ʹ); *HPRT* (5ʹ-ACACTG GCAAAACAATGCAGA-3ʹ and 5ʹ-ATCCAACAC TTCGTGGGGTC-3ʹ) or *TBP* (5ʹ-AAAATGGTG TGCACAGGAGC-3ʹ and 5ʹ-CTTCACATCACAGC TCCCCA-3ʹ), respectively. A real-time qPCR was performed using the BioRad iQ5 multicolor PCR detection system.

### Chromatin fractionation

The chromatin fractionation was performed as previously described [70] with some modifications. Briefly, cisplatin-treated HeLa cells were washed two times with PBS, trypsinized and collected in 1.5 ml tubes. The pellets were resuspended in buffer A (10 mM HEPES, pH 7.3, 50 mM NaCl, 0.3 M sucrose, 0.5% Triton X-100 and 1× protease inhibitor cocktail (Roche)). After incubation on ice for 15 min, the cytosolic fraction was separated by centrifugation at 4000 rpm for 5 min. The nuclear pellet was resuspended in buffer B (10 mM HEPES, pH 7.3, 200 mM NaCl, 1 mM EDTA, 0.5% NP40 and 1× protease inhibitor cocktail). After incubation on ice for 10 min, the nuclear fraction was separated by centrifugation at 13000 rpm for 2 min. The pellets were sonicated at 21% amplitude on ice for 60 sec pulse in buffer C (10 mM HEPES, pH 7.3, 500 mM NaCl, 1 mM EDTA, 0.5% NP40 and protease inhibitor cocktail). After centrifugation at 13000 rpm for 1 min at 4°C, the chromatin-bound protein (supernatant) was obtained and analysed by immunoblotting with the indicated antibodies.

### Western blotting

The cells were harvested and lysed using RIPA buffer (50 mM Tris-HCl, pH 8.0, 250 mM NaCl, 0.1% SDS, 0.02% sodium azide and 1% NP40). The protein samples (15 μg) were subjected to electrophoresis on denaturing 8% polyacrylamide gels and transferred to polyvinylidene difluoride membranes (Millipore Corp., Bedford, MA, USA). The blots were blocked with 3% bovine serum albumin in TBST buffer (50 mM Tris-HCl, pH 8.0, 150 mM NaCl and 0.1% Tween 20) for 3 h at 4°C. Subsequently, the blots were separately incubated with a primary antibody (obtained from BD Biosciences, San Jose, CA, USA/Santa Cruz Biotechnology Inc., Dallas, TX, USA/Cell Signalling Technology, Danvers, MA, USA) at the indicated dilutions: Rad50 (1:500), Mre11 (1:2000), Nbs1 (1:1000), Rad51 (1:500), α-tubulin (1:6000), Orc2 (1:500), β-catenin (1:5000), FANCD2 (1:100), CHK1 (1:100), GSK3β (1:1000), CHK1-p (1:1000) and γ-H2AX (1:1000) for 12 h at 4°C. The blots were washed 3 times with TBST buffer for 10 min each and incubated with horseradish peroxidase-conjugated secondary antibody (1:6000 dilution) for 4 h at 4°C. After washing the blots with TBST buffer thrice for 5 min each, the signals were visualized with the ImageQuant LAS 4000 system (GE Healthcare Life Sciences Imaging System).

### Green fluorescent protein-based DSB repair assay

The green fluorescent protein (GFP)-based assay for HR was performed as described [43]. Typically, U2OS cells were transfected with 16 μg of I-SceI plasmid DNA (per million cells) using a Bio-Rad gene pulsar X cell (250 V and 950 μF). Five hours following transfection, the cells were incubated separately with either DMSO, mirin, CHIR, FH535, LiCl, or in combination, as indicated in the figure legends. Forty-eight hour later, the cells were collected by trypsinization and analysed for GFP^+^ cells through fluorescence-activated cell sorting (FACS) analysis. In these assays, the efficiency of transfection of plasmid DNA was about 65–80%. The data obtained from the spontaneous GFP^+^ cells was subtracted from the frequency of I-SceI induced GFP^+^ cells.

### Data processing and statistical analyses

The *p value*s were calculated by the Student’s unpaired *t-test* as mentioned in the figure legends: ^*^ *p* < 0.05; ^**^ *p* < 0.01; ^***^ *p* < 0.001. ns, non-significant (*p* ≥ 0.05). Graphical display and statistical analyses of data were performed using GraphPad Prism software ver. 6.0. The group data are reported as mean ± S. E. M. of *n* = 3 independent experiments.

## CONCLUSIONS AND PERSPECTIVES

In this study, we sought to elucidate the potential relationship between Wnt/β-catenin signalling and the MRN complex in ICL repair. Consistent with the established roles of the MRN complex subunits in sensing, detecting and processing of DSBs during HR-directed repair, a marked increase in the abundance of MRN complex subunits occurred following exposure to cisplatin. Our studies also show that promoting Wnt/β-catenin signalling, after cells were exposed to cisplatin, reduces the cell survival due to cell cycle progression of cells bearing unresolved DNA damage, increases the number of γH2AX foci-positive cells, and inhibits HR. Conversely, inhibition of Wnt/β-catenin signalling resulted in increased cell viability. Although inhibition of Wnt/β-catenin signalling led to an unexpected decrease in HR efficiency, it resulted in increased cell survival, enhanced cell cycle progression, and reduced the number of cells having γ-H2AX-positive foci. These results suggest that Wnt/β-catenin signalling plays a pivotal role in cisplatin-induced DNA damage and repair, indicating a crosstalk between Wnt/β-catenin signalling and MRN mediated repair of ICL DNA damage.

What do these new insights convey about the functional relationship between Wnt/β-catenin signalling and the MRN complex in the regulation of ICL DNA repair? This study demonstrates that the MRN complex subunits are targets of the Wnt/β-catenin signalling pathway. Although the MRN complex is key for the repair of DSBs, the observed repair was dependent on the Wnt/β-catenin signalling pathway. The perturbation of Wnt/β-catenin signalling by loss or gain of function has been linked to the progression of various diseases, including cancer. Several strategies are currently being used to enhance the sensitivity of tumour cells to cisplatin. Clinically, the functional characterization of the critical Wnt/β-catenin signalling axis in ICL DNA repair may shed light on novel therapeutic modalities. In that scenario, a combinatorial treatment of chemotherapeutic agents along with small molecule inhibitors of Wnt signalling seems very promising. However, it is possible that there exist additional downstream targets of Wnt/β-catenin signalling that may be functionally important in conferring cisplatin resistance; this remains to be determined. Overall, this study demonstrates a novel mechanism whereby Wnt/β-catenin signalling regulates ICL repair and, consequently, genome stability and cell proliferation.

## SUPPLEMENTARY MATERIALS



## Abbreviations

DMSO: dimethyl sulfoxide
DSBs: double strand breaks
FACS: fluorescence-activated cell sorting
FBS: fetal bovine serum
ICL: interstrand cross-links
MTT: 3-(4,5-dimethylthiazol-2-yl)-2,5-diphenyltetrazolium bromide
PAGE: polyacrylamide gel electrophoresis
PBS: phosphate buffered saline
RT-qPCR: reverse transcription quantitative polymerase chain reaction
SDS: sodium dodecyl sulphate
