# Supplementary material for: Evidence for functional and regulatory cross-talk between Wnt/β-catenin signalling and Mre11–Rad50–Nbs1 complex in the repair of cisplatin-induced DNA cross-links
Source: Oncotarget. 2020 Nov 3;11(44):4028–44. doi: 10.18632/oncotarget.27777 (PMC7646826; doi:10.18632/oncotarget.27777)
Supplement: Supplementary file 1 [file oncotarget-11-4028-s001.pdf]

Evidence for functional and regulatory cross-talk between Wnt/β-catenin signalling and Mre11–Rad50–Nbs1 complex in the repair of cisplatin-induced DNA cross-links

SUPPLEMENTARY MATERIALS

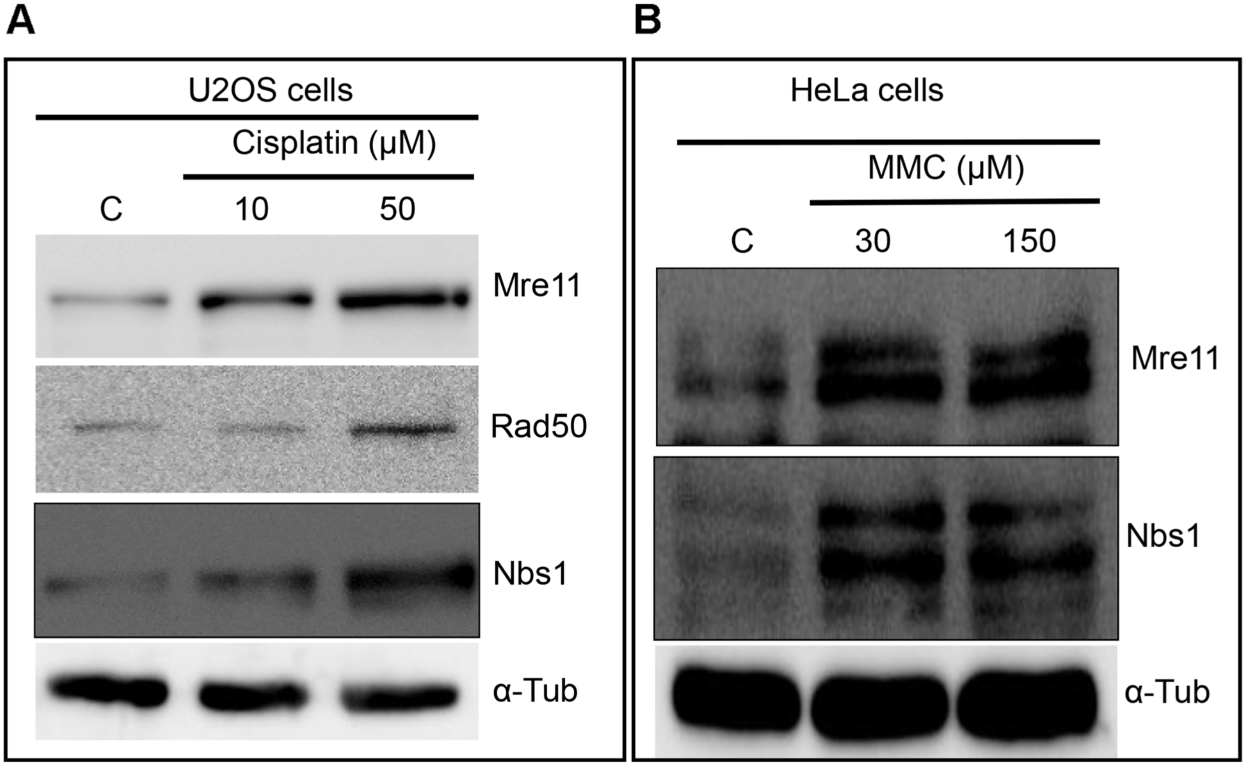

Supplementary Figure 1: (**A**) Western blots showing dose dependent enrichment of MRN complex in U2OS cells after treatment with indicated dose of cisplatin for 3 h followed by 12 h recovery in fresh medium. (**B**) Increase in the abundance of Mre11 and Nbs1 proteins in HeLa cells following treatment with increasing dose of mitomycin C (MMC) continuously for 12 h.

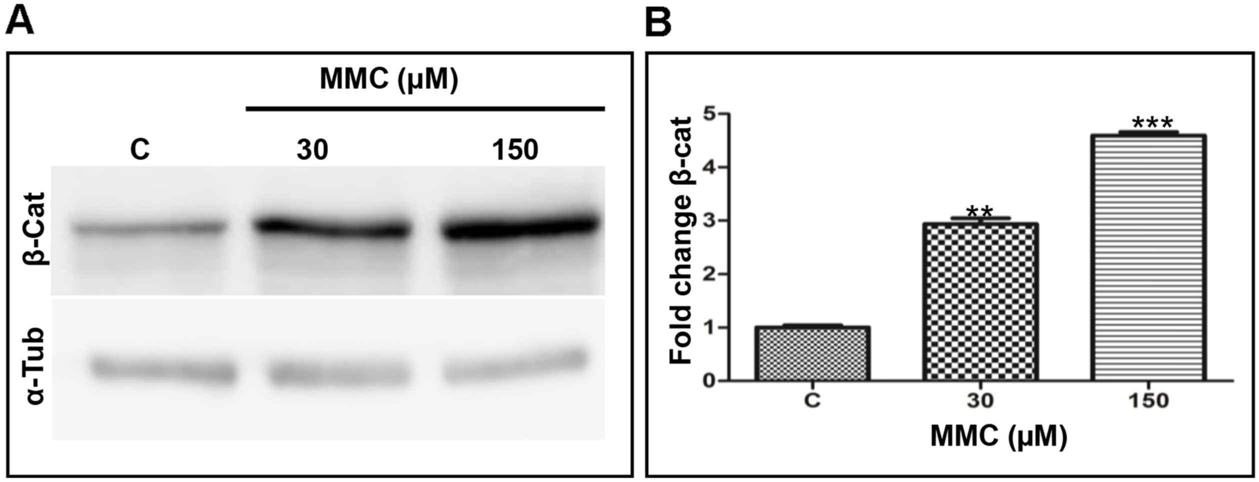

Supplementary Figure 2: Mitomycin C induces the expression of β-catenin. (**A**) Western blot analyses showing the levels of β-catenin in U2OS cells after treatment with increasing concentrations of mitomycin C for 16 h. (**B**) Histograms show the mean values of the relative level of β-catenin as determined by Western blotting, normalized to the level of α-tubulin. The data shows mean ± SEM of three independent experiments. Data is analyzed by student's *t*-test and significant differences are denoted by $^{*}p < 0.05$; $^{**}p < 0.01$; $^{***}p < 0.001$. ns, not significant.

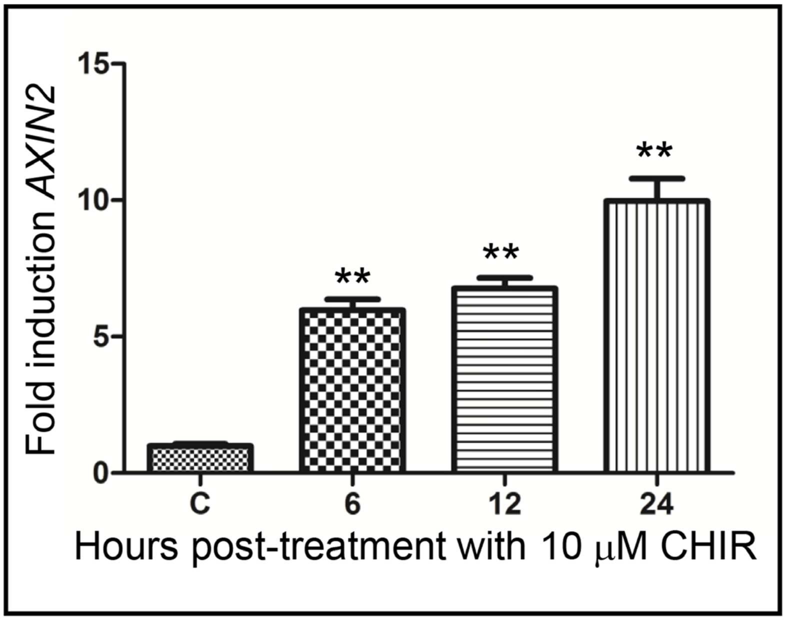

Supplementary Figure 3: Analysis of *AXIN2* mRNA levels following treatment with CHIR. The histograms represent mean ± SEM from three independent experiments normalized to those of the untreated cells. Statistical analysis was done by student's *t*-test. $^{*}p < 0.05$; $^{**}p < 0.01$; $^{***}p < 0.001$. ns, not significant.

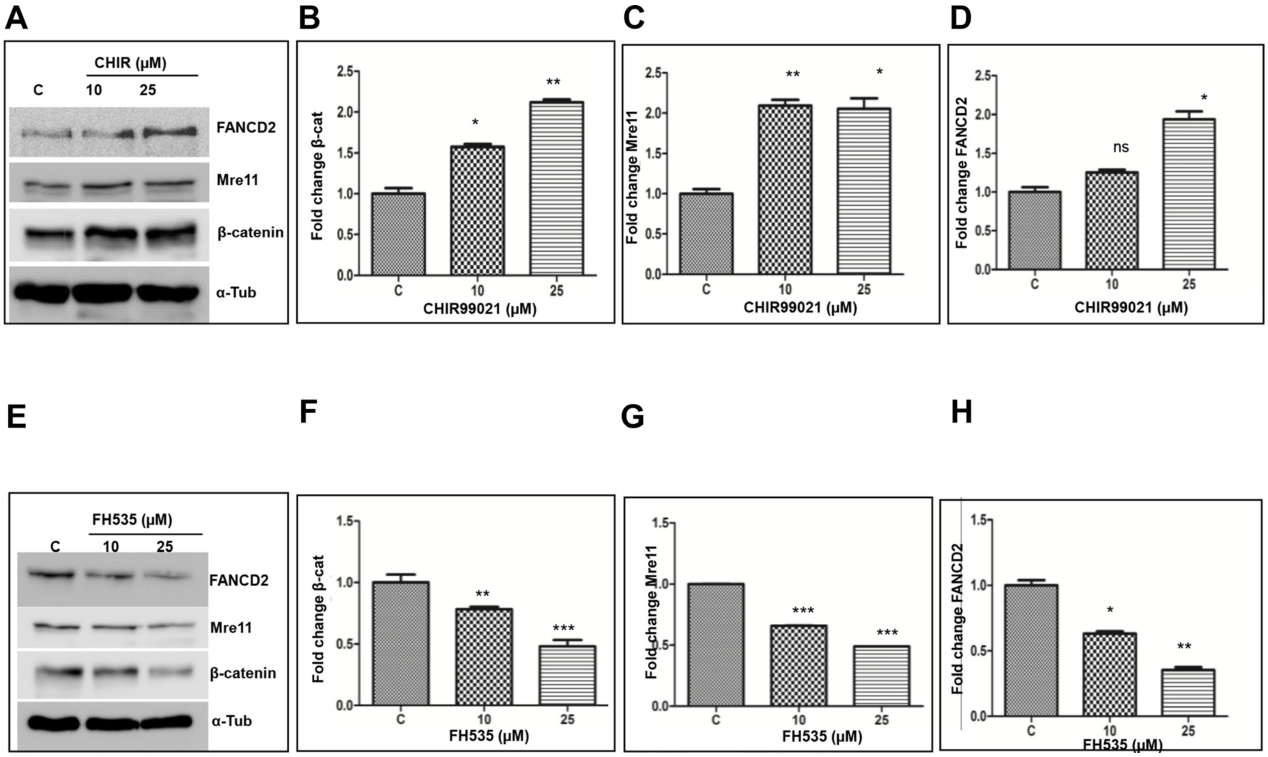

Supplementary Figure 4: CHIR stimulates the expression of β-catenin, Mre11 and FANCD2. (**A**) Immunoblots showing increased expression of β-catenin, Mre11 and FANCD2 following inhibition of GSK3β by CHIR. The whole cell lysates were analyzed 24 h after CHIR treatment. (**B–D**) Histograms show the mean values of relative level of FANCD2, Mre11and β-catenin as determined by western blotting. (**E**) Immunoblots showing the levels of β-catenin, Mre11 and FANCD2 following treatment with increasing dose of FH535. The whole cell lysates were analyzed 24 h after treatment. (**F–H**), Histograms show the mean values of relative level of FANCD2, Mre11and β-catenin as analyzed by western blotting. α-tubulin was used as an internal control and normalization. Statistical analysis was done by student's *t*-test. $n = 3$; $^{*}p < 0.05$; $^{**}p < 0.01$; $^{***}p < 0.001$; ns, not significant.

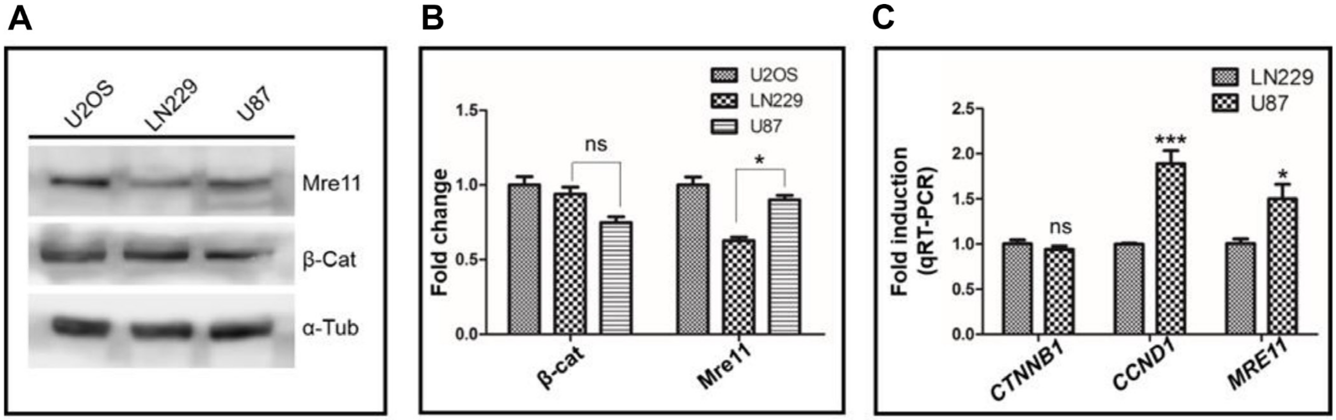

Supplementary Figure 5: Mre11 expression pattern in cell lines LN229 and U87 with altered Wnt/β-catenin signalling. (**A**) Western blot analysis depicting the levels of β-catenin and Mre11 in U2OS, and glioma cell lines LN229 and U87. (**B**) Histogram shows the mean values of relative level of β-catenin and Mre11 as determined by western blotting. α-tubulin was used for loading control and normalization. (**C**) RT-qPCR data showing differential expression profiles of *CTNNB1* (β-catenin), *CCND1* (Cyclin D1) and *MRE11* genes in human glioma cell lines LN229 and U87. The RT-qPCR normalization factor is based on the geometric mean of relative amounts of three control genes: *GAPDH*, *HPRT* and *TBP*. The standard deviations were derived from two independent experiments. Statistical analysis was done by Student's *t*-test. $n = 3$; $^{*}p < 0.05$; $^{**}p < 0.01$; $^{***}p < 0.001$. ns, not significant.

Supplementary Table 1: LC_{50} values for U2OS cells treated with cisplatin alone or in combination with mirin and CHIR99021 or FH535

**Compounds**	**LC_{50} (µM)**
Cisplatin alone	17.1 ± 0.87
Cisplatin + Mirin	11.98 ± 1.9
Cisplatin + CHIR99021 (↑)	8.96 ± 0.72**
Cisplatin + FH535 (↓)	28.27 ± 7.94

The arrows in parenthesis indicate upregulation (↑) or downregulation (↓) of Wnt signalling. Data is analyzed by student's *t*-test and significant differences are denoted by $^{*}p < 0.05$; $^{**}p < 0.01$; $^{***}p < 0.001$. The data represent the results (mean ± SEM) of three independent experiments.

Supplementary Table 2: LC_{50} values for long term survival of U2OS cells under different experimental conditions

Compounds	LC_{50} (μM)
Cisplatin alone	19.36 ± 0.43
Cisplatin + Mirin	16.05 ± 0.74*
Cisplatin + CHIR99021 (↑)	13.03 ± 0.78**
Cisplatin + FH535 (↓)	24.24 ± 1.38*

The arrows in parenthesis indicate upregulation (↑) or downregulation (↓) of Wnt signalling. The values represent mean ± SEM of three independent experiments. Data is analyzed by student's *t*-test and significant differences are denoted by $^{*}p < 0.05$; $^{**}p < 0.01$; $^{***}p < 0.001$.

Supplementary Table 3: LC_{50} values for long term survival of U2OS cells which were pre-treated with indicated compounds prior to addition of cisplatin

Compounds	LC_{50} (μM)
Cisplatin alone	16.99 ± 0.50
Mirin + Cisplatin	8.49 ± 1.54**
CHIR99021 (↑) + Cisplatin	6.43 ± 0.62***
FH535 (↓) + Cisplatin	20.30 ± 0.65*

The values represent mean ± SEM of three independent experiments. Data is analyzed by student's *t*-test and significant differences are denoted by $^{*}p < 0.05$; $^{**}p < 0.01$; $^{***}p < 0.001$. The arrows pointing upward and downward indicate Wnt promoting or inhibiting molecules, respectively.

Supplementary Table 4: The percentage of cells at different phases of the cell cycle following treatment with cisplatin alone or in combination with CHIR or FH535

Treatment	G1	S	G2/M
Control	58.695 ± 0.985	23.67 ± 0.68	17.635 ± 0.305
Cisplatin	8.41 ± 0.46***	14.535 ± 0.255**	77.055 ± 0.715***
Cisplatin+ CHIR	0.295 ± 0.025***	99.705 ± 0.025***	–
Cisplatin+ FH535	30.485 ± 0.215**	27.595 ± 1.555	41.92 ± 1.34**

The values represent mean ± SEM of three independent experiments. Data is analyzed by student's *t*-test and significant differences are denoted by $^{*}p < 0.05$; $^{**}p < 0.01$; $^{***}p < 0.001$.

Supplementary Table 5: The percentage of cells at different phases of the cell cycle following treatment with CHUR or FH535

Treatment	G1	S	G2/M
Control	61.525 ± 0.335	23.845 ± 0.695	14.63 ± 1.03
CHIR99021	48.2 ± 2.23*	33.37 ± 0.15**	18.43 ± 2.38
FH535	71.66 ± 0.59**	16.555 ± 0.095**	11.785 ± 0.685

The values represent mean ± SEM of three independent experiments. Data is analyzed by student's *t*-test and significant differences are denoted by $^{*}p < 0.05$; $^{**}p < 0.01$; $^{***}p < 0.001$.
